# Exploring the enigma: history, present, and future of long non-coding RNAs in cancer

**DOI:** 10.1007/s12672-024-01077-y

**Published:** 2024-06-07

**Authors:** Qais Ahmad Naseer, Abdul Malik, Fengyuan Zhang, Shengxia Chen

**Affiliations:** Department of Laboratory Medicine, School of Medicine, Jiangsu University, 301 Xuefu Road, Zhenjiang, 212013 China

**Keywords:** LncRNA, Gene expression, Biogenesis, Regulatory mechanisms, Genomic location

## Abstract

Long noncoding RNAs (lncRNAs), which are more than 200 nucleotides in length and do not encode proteins, play crucial roles in governing gene expression at both the transcriptional and posttranscriptional levels. These molecules demonstrate specific expression patterns in various tissues and developmental stages, suggesting their involvement in numerous developmental processes and diseases, notably cancer. Despite their widespread acknowledgment and the growing enthusiasm surrounding their potential as diagnostic and prognostic biomarkers, the precise mechanisms through which lncRNAs function remain inadequately understood. A few lncRNAs have been studied in depth, providing valuable insights into their biological activities and suggesting emerging functional themes and mechanistic models. However, the extent to which the mammalian genome is transcribed into functional noncoding transcripts is still a matter of debate. This review synthesizes our current understanding of lncRNA biogenesis, their genomic contexts, and their multifaceted roles in tumorigenesis, highlighting their potential in cancer-targeted therapy. By exploring historical perspectives alongside recent breakthroughs, we aim to illuminate the diverse roles of lncRNA and reflect on the broader implications of their study for understanding genome evolution and function, as well as for advancing clinical applications.

## Background: the emergence of long noncoding RNAs

**Fig. 1:**
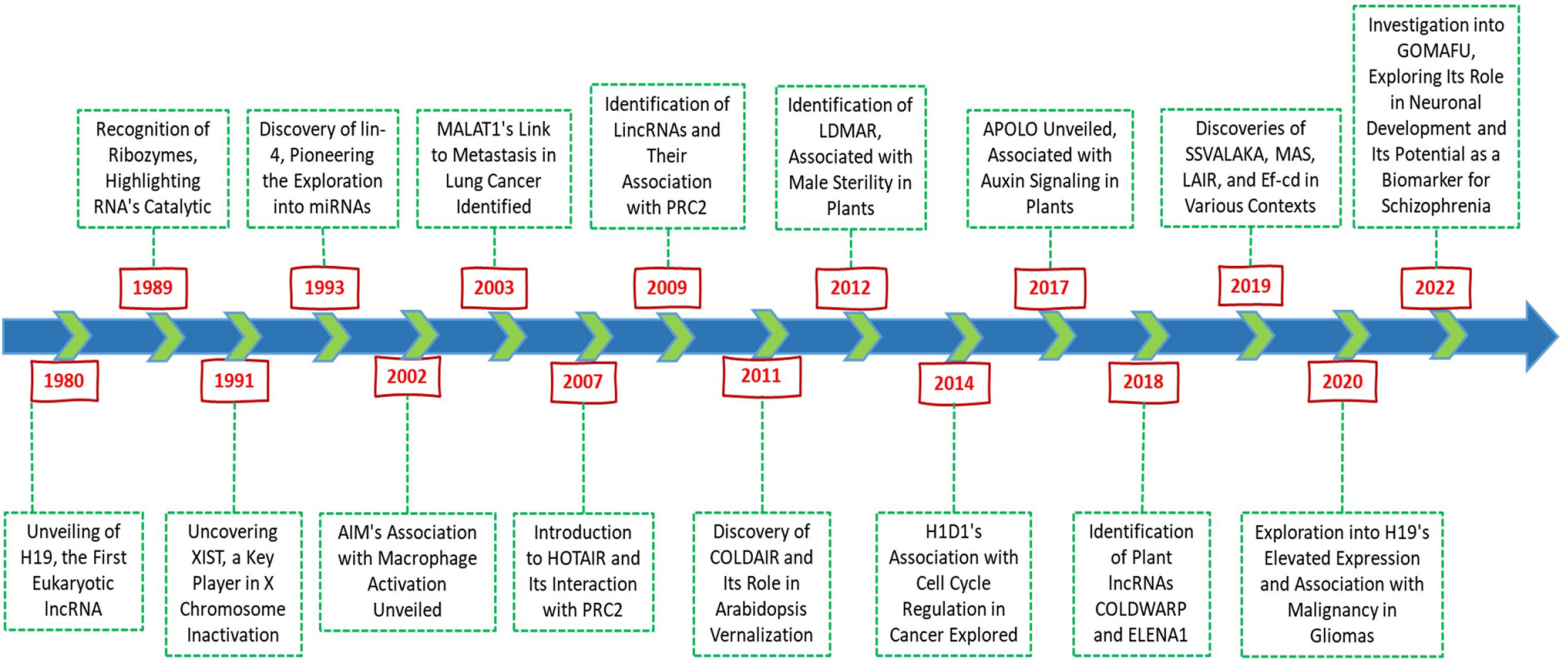
Timeline of key discoveries in lncRNA research from 1980 to 2022

Long noncoding RNAs (lncRNA) have become integral to our understanding of genome evolution, size, and complexity, a process that began in the 1950s. The investigation of the C-value, which measures the DNA content of a haploid genome, revealed intriguing paradoxes, notably the limited correlation between DNA quantity and organismal complexity [1, 2]. This “C-value paradox” sparked widespread discussion, particularly with discoveries that some organisms, considered evolutionarily “simpler,” possess genomes significantly larger than those of “higher” organisms, including humans [3, 4]. A pivotal moment in resolving this paradox came with the realization that a substantial fraction of the genome is noncoding and not dedicated to protein synthesis [5, 6]. Initially, termed “junk DNA,” these noncoding regions, which make up 50–70% of the human genome, were later found to play crucial roles beyond their initial dismissal as genomic filler [6–11]. The 1970s marked the beginning of a shift in perspective with the observation of “pervasive transcription,” indicating that a vast portion of the genome, beyond known coding regions, is transcribed. These RNAs include not only coding genes but also heterogeneous nuclear RNAs (hnRNAs) and other RNA types, such as rRNA and tRNA [6, 12, 13]. The late 1990s and early 2000s brought technological advancements that further illuminated the genome’s transcriptional landscape, such as the recognition of ribozymes in 1989, which highlighted the catalytic capabilities of RNA, and the discovery of XIST in 1991, which provided a new understanding of X chromosome inactivation. The subsequent discovery of lin-4 in 1993 pioneered the exploration of microRNAs (miRNAs), a class of small noncoding RNAs that, similar to lncRNAs, are involved in the posttranscriptional regulation of gene expression [14–18]. Early hypotheses by Jacob and Monod and later by Britten and Davidson proposed regulatory roles for noncoding RNAs, suggesting their involvement in gene expression modulation and signal transmission [19, 20]. The discovery of lncRNAs such as H19 and Xist in the early 1990s underscored their significance in epigenetic regulation [21–24]. Challenging the notion of “transcriptional noise” and sparking debates about the functional relevance of these transcripts [25–28]. The ongoing exploration of lncRNA is progressively unveiling the complex mechanisms of gene expression regulation, revealing a complex landscape of genomic functionality that extends significantly beyond the coding sequences traditionally emphasized in molecular biology. This burgeoning field of research, as illustrated in Fig. 1, promises to substantially deepen our understanding of genome architecture and its regulation, implicating the expression of lncRNAs in pivotal developmental stages, physiological conditions, and a spectrum of pathologies, notably cancer.

## Complexity and diverse functional implications in genomic contexts

### General features of lncRNA

LncRNAs, which are characterized by a length surpassing 200 nucleotides and lack protein-coding capability, are predominantly transcribed by RNA polymerase II. This mirrors the intricate and adaptable nature of mRNA in terms of structure, composition, and function [29, 30]. These molecules play pivotal roles in the intricate dance of genomic regulation, engaging with proteins, DNA, and RNA to orchestrate the organization of the genome, cellular architecture, and nuanced layers of gene expression regulation [29, 31–33]. Their influence extends from the nucleus to the cytoplasm, where they oversee critical processes such as translation, metabolism, and signal transduction, demonstrating a remarkable specificity to tissue types and developmental stages [34–37]. Among the diverse classes of lncRNAs, stand-alone lncRNA, or "lincRNA," represent unique transcription units that operate independently of protein-coding genes. These lncRNAs, including notable examples such as Xist, H19, HOTAIR, and MALAT1, are characterized by their transcription by RNA Pol II, subsequent polyadenylation, and splicing, typically spanning an average length of 1 kb [38–41]. This independence from coding sequences allows them to serve as crucial regulators of gene expression and chromatin architecture.

Natural antisense transcripts (NATs) add another layer of complexity, often forming sense‒antisense pairs with coding transcripts, such as Xist/Tsix and Kcnq1/Kcnq1ot1. Despite their abundance, the full extent of the biological functions of NATs remains an area ripe for exploration [42–46]. Similarly, pseudogenes, the genomic remnants once considered mere evolutionary artifacts, have revealed their capacity to influence gene expression, challenging our understanding of genomic ‘junk’ [47–49]. Intriguingly, long intronic ncRNAs, found within the introns of annotated genes, exhibit diverse expression patterns and have been implicated in specific biological processes, such as the regulation of plant vernalization by COLDAIR [50, 51]. Moreover, the genomic landscape was further enriched by divergent, promoter-linked, and enhancer-associated RNAs, including TSSa-RNAs, uaRNAs, and PROMPTs. Although these short transcripts are often rapidly degraded, their production at transcription start sites and enhancers hints at potential regulatory roles yet to be fully understood [52–58].

### Functions and mechanisms of lncRNA

**Fig. 2:**
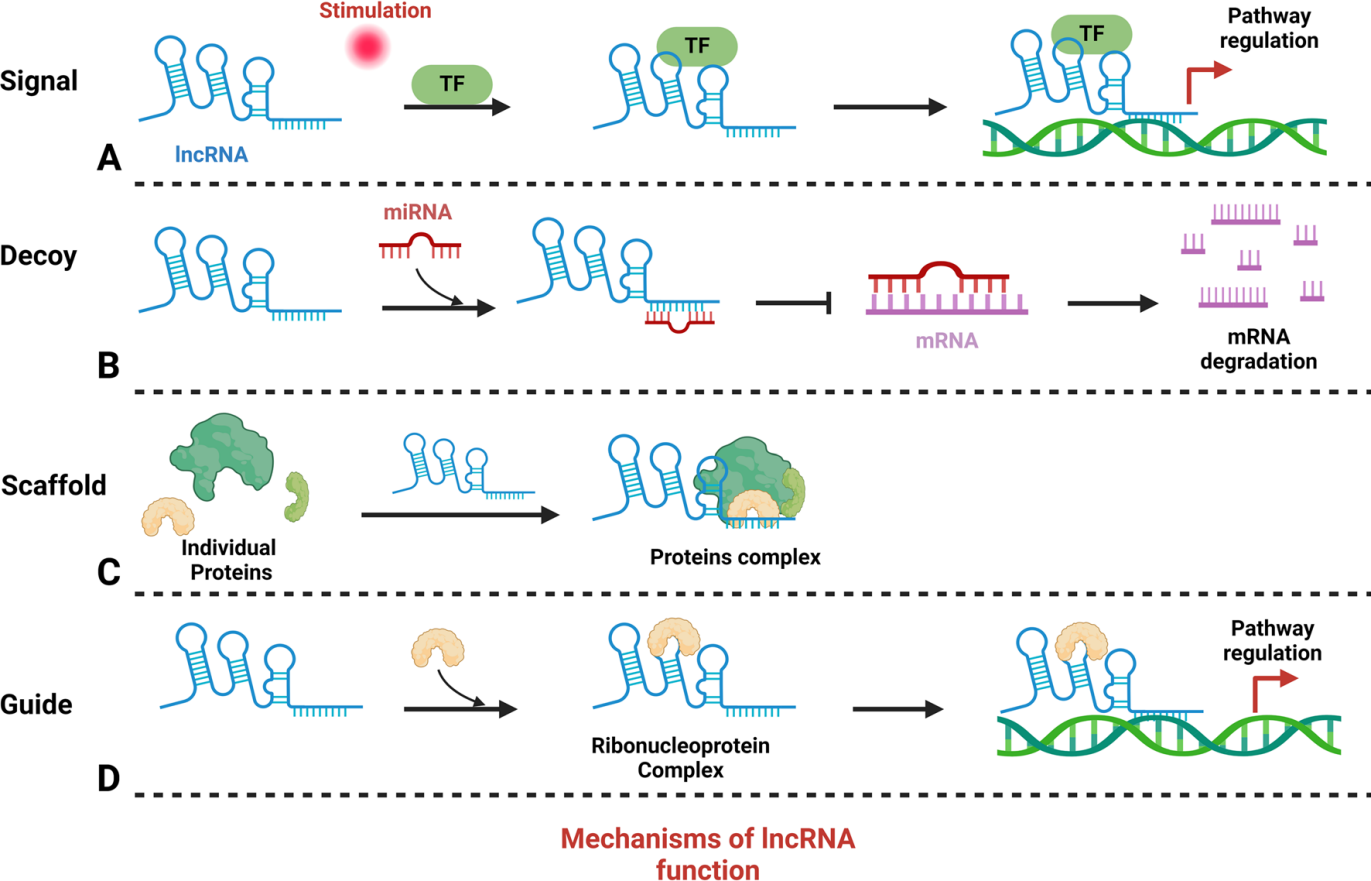
Functional mechanisms of lncRNA **A** In response to diverse stimuli, lncRNA regulates signaling pathways by binding to transcription factors. **B** Acting as miRNA sponges, lncRNA inhibits mRNA degradation. **C** Serving as scaffolds, lncRNA aids in forming protein complexes, thereby regulating target gene transcription. **D** LncRNA also guides ribonucleoprotein complexes to specific DNA sequences, influencing gene expression

Insights into the multifaceted mechanisms of lncRNA are pivotal for elucidating their roles in diseases, particularly cancer. Studies have highlighted the integration of lncRNAs into cellular molecular networks, underscoring their importance in developing cancer therapies [59–62]. Table 1 categorizes a spectrum of lncRNAs associated with various cancers, delineating their unique roles and actions. These lncRNAs are intricately regulated, integral to the complex network of cellular regulation, and instrumental to both disease pathogenesis and normal physiological processes. They interact with DNA, RNA, and proteins, significantly influencing gene expression (Fig. 2). As signals, certain lncRNAs, such as Xist, which is transcribed from the inactive X chromosome, indicate cellular states and trigger X chromosome inactivation [63, 64]. Scaffold lncRNA, such as HOTAIR, form structural bases for regulatory complexes, recruiting enzymes for chromatin modification and consequently altering gene expression [65]. Decoy lncRNA, such as PANDA, bind transcription factors, thereby preventing them from activating proapoptotic genes [66]. Competing with endogenous RNA (ceRNA), lncRNA can sponge miRNAs, protecting target mRNAs from degradation—a mechanism exhibited by LINC00680, which binds to miR-423-5p to modulate PAK6 expression in esophageal squamous cell carcinoma [67]. Guide lncRNA, exemplified by MEG3, direct transcriptional machinery to specific genomic regions, modulating gene expression patterns [68].

**Table 1:** Roles and regulatory mechanisms of lncRNAs across various cancer types

lncRNA	Cancer type	Role/function	Mechanism of regulation	Ref
TUG1	Bladder cancer	TUG1 promotes BC cell proliferation, migration, and invasion	Modulate miR-145/ZEB2 axis	[169]
TUG1	Bladder cancer	Migration and invasion of T24 and EJ cells	Promote HMGB1 expression	[170]
HOTAIR	Breast cancer	Promotes tumorigenic activity in breast CSCs	Facilitate HSPA1A expression via sequestering miR-449b-5p	[171]
LINC00511	Breast cancer	Enhanced cellular proliferation, increased sphere-formation capability, elevated expression of stem factors (Oct4, Nanog, SOX2), and heightened tumor growth were observed	Modulate miR-185/STXBP4	[172]
LINC02582	Breast cancer	Promoted radioresistance	Interact with USP7 to deubiquitinate and stabilize CHK1	[173]
LINC00963	Breast cancer	Stimulates the proliferation and tumorigenesis of breast cancer cells Inhibits DNA damage and oxidative stress while increasing the sensitivity of breast cancer cells to radiation	Modulate miR-324-3p/ACK1 axis	[174]
HOTAIR	Breast cancer	Facilitates the proliferation of breast cancer cells by modulating the cell cycle and apoptosis. Additionally, it enhances DNA repair mechanisms and contributes to radioresistance	Modulate EZH2	[175]
H19	Breast cancer	Suppression resulted in the inhibition of proliferation, migration, and invasion, coupled with the induction of apoptosis	Modulate miR-130a-3p/SATB1	[176]
TRPM2-AS	Gastric cancer	Promote the proliferation, metastasis and radioresistance	Enhance the expression of FOXM1 by acting as a sponge of miR-612	[177]
NEAT1	Gastric cancer	Enhanced radio-sensitivity	Modulate miR-27b-3p	[178]
PCAT1	Cervical cancer	The enhancement of radiosensitivity in CC cells through silencing is evident in reduced proliferation, migration, and invasion	Modulate miR-128/GOLM1 axis	[179]
LINC00958	Cervical cancer	Suppresses the proliferation and tumor growth of cervical cancer cells, concurrently fostering apoptosis in these cells	Modulate miR-5095/RRM2	[180]
GAS5	Cervical cancer	Enhanced radio-sensitivity	Modulate miR-106b/IER3 axis	[181]
HOTAIR	Cervical cancer	Nullified the impact of radiation on cell viability and apoptosis in the cells	Promote HIF-1α expression	[182]
HOTAIR	Cervical cancer	Suppressed apoptosis and facilitated cellular proliferation, progression of the cell cycle, migration, and invasion, leading to the induction of radioresistance	Regulate p21 expression	[183]
HOTAIR	Colorectal cancer	Knockdown weakened cell viability, induced cell apoptosis, inhibited cell autophagy, and enhanced cell radiosensitivity	Regulate microRNA-93/ATG12	[184]
Lnc-RI	Colorectal cancer	Silencing inhibited cell growth and promoted apoptosis rates, increased radiosensitivity	Regulate NHEJ repair through miR-4727-5p/LIG4	[185]
LINC00958	Colorectal cancer	Stimulated in vitro cell proliferation while inhibiting apoptosis and reducing sensitivity to radiotherapy. Additionally, it facilitated tumor growth in in vivo experiments	Modulate miR-422a/MAPK1 axis	[186]
EGOT	Rectal cancer	Knockdown inhibited the proliferation and colony formation, induced the apoptosis and improved the radiosensitivity	Modulate miR-211-5p/ErbB4 axis	[187]
MAGI2-AS3	Esophageal cancer	Promoted cell apoptosis and inhibited proliferation and radio-resistance	Down-regulate HOXB7 through interaction with EZH2	[188]
DNM3OS	Esophageal squamous cell cancer	Enhance radioresistance	Regulate DNA damage response	[189]
FAM201A	Esophageal squamous cell cancer	Enhanced the radiosensitivity	Regulate ATM and mTOR expression via miR-101	[190]
NORAD	Esophageal Squamous cell cancer	Knockdown sensitizes ESCC cells to radiation treatment	Modulate EEPD1/ATR/Chk1 axis and inhibit pri-miR-199a1	[191]
TUG1	Esophageal squamous cell cancer	Knockdown inhibition proliferation and colony formation and induced apoptosis	Modulate miR-144-3p and MET/EGFR/AKT axis	[192]
MALAT1	Esophageal squamous cell cancer	Suppressed the reduction in cell viability induced by irradiation and increased the occurrence of apoptosis	Modulate Cks1 expression	[193]
HOTAIRM1	Glioblastoma	Knockdown reduced cell viability, decreased invasive growth and diminished colony formation capacity	Regulate mitochondrial function and ROS levels via TGM2	[194]
HMMR-AS1	Glioblastoma	Knockdown inhibits cell proliferation, migration, invasion, and mesenchymal phenotypes	Regulate DNA repair proteins ATM, RAD51, and BMI1	[195]
RBPMS-AS1	Glioblastoma	Enhanced the radiosensitivity and cell apoptosis while suppressing proliferation	Promote NRGN transcription through the miR-301a-3p/CAMTA1 axis	[196]
TPTEP1	Glioma	Weakened the stemness and radioresistance of glioma in both laboratory and animal testing settings	Stimulate the P38 MAPK signaling through interacting with miR‑106a‑5p	[197]
linc-RA1	Glioma	Promoted glioma radioresistance in vitro and in vivo	Prevent H2Bub1/USP44 combination	[198]
TP53TG1	Glioma	Promoted the proliferation, colony formation, autophagy, and radioresistance, and restrained the apoptosis	Modulate miR-524-5p/RAB5A axis	[199]
SNHG18	Glioma	Suppressed the radioresistance	Inhibit semaphorin 5A	[200]
NCK1-AS1	Glioma	Knockdown impedes cell proliferation and amplifies cell apoptosis	Modulate miR-22-3p/IGF1R	[201]
LincRNA-p21	Glioma	N/A	Regulate β-catenin	[202]
LINC01123	Glioma	The knockdown resulted in diminished cell proliferation, impaired colony formation capabilities, and heightened susceptibility to apoptosis following 4 Gy X-ray irradiation	Modulate miR-151a/CENPB axis	[203]
XIST	Glioma	Knockdown impeded cell proliferation, curtailed invasion, and prompted cell apoptosis by increasing cell sensitivity to X-ray radiation	Modulate miR-329-3p/CREB1 axis	[204]
LINC00520	Head and neck squamous cell cancer	Silencing enhanced radiosensitivity and restrained cell proliferation, invasion, migration, and apoptosis	Modulate miR-195/HOXA10	[205]
HOTAIR	Laryngeal cancer	Reduced laryngeal cancer cell radiosensitivity	Modulate miR-454-3p/E2F2 axis	[206]
DGCR5	Laryngeal cancer	Silencing inhibited the stemness and enhance the radiosensitivity	Modulate miR-506/Wnt pathway	[207]
DGCR5	Laryngeal cancer	Promoted cell proliferation and radioresistance	Regulate miR-195	[208]
NEAT1	Nasopharyngeal cancer	Suppressed cell growth, invasion, and radiation resistance in laboratory settings and inhibited tumor metastasis in animal studies	Modulate miR-101-3p/EMP2 axis	[209]
PVT1	Nasopharyngeal cancer	The knockdown resulted in decreased cell proliferation, diminished colony formation, reduced tumorigenesis, and heightened radiosensitivity	Stabilize HIF-1α through promoting the binding between H3K9ac and TIF1β	[210]
linc00312	Nasopharyngeal cancer	Enhance the responsiveness of cells to ionizing radiation	Target DNA-PKcs and impairing DNA damage repair	[211]
LINC00114	Nasopharyngeal cancer	Knockdown inhibited proliferation, migration, and radioresistance	Regulate ERK/JNK signaling pathway via targeting miR-203	[212]
PVT1	Nasopharyngeal cancer	Knockdown inhibited cell proliferation, radioresistance and promoted cell apoptosis	Modulate miR-515-5p/PIK3CA	[213]
CASC19	Nasopharyngeal cancer	Knockdown enhanced apoptosis, radiosensitivity, and suppressed cellular autophagy	Promote autophagy via AMPK-mTOR pathway	[214]
ANCR	Nasopharyngeal cancer	The knockdown impeded the growth of NPC cells and reduced their resistance to radiation	Inhibit PTEN expression	[215]
LINC-PINT	Nasopharyngeal cancer	Demonstrated responsiveness to DNA damage and decreased cellular tolerance to ionizing radiation both in laboratory experiments and animal models	Inhibit DNA damage repair through ATM/ATR-Chk1/Chk2	[216]
PTPRG-AS1	Nasopharyngeal cancer	The silencing heightened sensitivity to radiotherapy and induced cell apoptosis, concurrently inhibiting cell migration, invasion, and proliferation	Modulate miR-194-3p/PRC1	[217]
MALAT1	Nasopharyngeal cancer	Knockdown sensitize to radiation	Modulate miR-1/slug axis	[218]
ROR	Hepatocellular cancer	The knockdown diminishes the radiosensitivity of parental HCC cells both in vitro and in vivo by reducing their DNA repair capacity	Modulate miR-145/RAD18 axis	[219]
GAS5	Hepatocellular cancer	Enhanced the radiosensitivity	Modulate miR-144-5p/ATF2 axis	[220]
MIR22HG	Hepatocellular cancer	Increase radiosensitivity	Promote the production of miR-22-5p	[221]
LINC00483	Lung cancer	Silencing inhibited cell proliferation, migration, invasion and enhanced radiosensitivity	Modulate miR-144/HOXA10	[222]
HOTAIR	Lung cancer	Reduced radio-sensitivity	Regulate β-catenin	[223]
AGAP2-AS1	Lung cancer	Knockdown reduced radioresistance	Modulate miR-296/NOTCH2	[224]
LINC00461	Lung cancer	Knockdown promoted cell proliferation, migration and invasion, and enhanced radiosensitivity	Modulate miR-195/HOXA10	[225]
KCNQ1OT1	Lung adenocarcinoma	Promoted autophagy and radioresistance	Induce ATG5/ATG12-mediated autophagy via miR-372-3p	[226]
SBF2-AS1	Non-small cell lung cancer	Knockdown promoted apoptosis and inhibited proliferation and radioresistance,	Modulate microRNA-302a/MBNL3 axis	[227]
FAM201A	Non-small cell lung cancer	Knockdown inhibited proliferation, enhanced apoptosis and radiosensitivity	Upregulate EGFR and HIF-1α via miR-370	[228]
PVT1	Non-small cell lung cancer	Knockdown inhibited proliferation, enhanced apoptosis and radiosensitivity	Regulate miR-195	[229]
CYTOR	Non-small cell lung cancer	Reduces radiosensitivity	Modulate miR-206/PTMA axis	[230]
CBR3-AS1	Non-small cell lung cancer	knockdown reduced proliferation, invasion, and migration; inhibited cell cycle progression; and promoted apoptosis, radiosensitivity	Modulate miR-409-3p/SOD1	[231]
HNF1A-AS1	Non-small cell lung cancer	Knockdown inhibited proliferation, enhanced apoptosis and radiosensitivity	Modulate miR-92a-3p/MAP2K4/JNK axis	[232]
Linc-SPRY3	Non-small cell lung cancer	Knockdown increased survival and reduced apoptosis	Interact with IGF2BP3 and affect RNA stability in HMGA2 and c-MYC mRNAs	[233]
GAS5	Non-small cell lung cancer	Suppressed cell proliferation and invasion, and enhanced radiosensitivity	Modulate miR-135b	[234]
RBM5-AS1	Medulloblastoma	Silencing inhibited proliferation and enhanced apoptosis radiosensitivity	Stabilization of SIRT6 protein	[235]
LINC00518	Melanoma	Knockdown inhibited cell invasion, migration, proliferation, and clonogenicity and enhanced radioresistance	Regulate glycolysis through an miR-33a-3p/HIF-1α negative feedback loop	[236]
LINC01224	Melanoma	Knockdown inhibited cell viability and proliferation but enhanced cell apoptosis and radiosensitivity	Modulate miR-193a-5p/NR1D2 axis	[237]
XIST	Neuroblastoma	Knockdown inhibited proliferation, enhanced radiosensitivity	Modulate the miR-375/L1CAM	[238]
LINC01410	Neuroblastoma	Knockdown inhibited proliferation and invasion, and increased radiosensitivity	Modulate miR-545-3p/HK2 axis	[239]
HULC	Prostate cancer	Knockdown promoted autophagy, apoptosis, and enhanced radiosensitivity	Modulate autophagy via Beclin-1 and mTOR	[240]
TUG1	Prostate cancer	Knockdown inhibited proliferation, enhanced cell apoptosis and radiosensitivity	Modulate miR-139-5p/SMC1A axis	[241]
GAS5	Prostate cancer	Suppressed cell viability and migration, enhanced radiosensitivity	Modulate miR-320a/RAB21 axis	[242]
LINC02532	Renal cell cancer	Knockdown enhanced cell radiosensitivity	Modulate miR-654-5p/YY1 axis	[243]
SNHG7	Thyroid cancer	Promote proliferation and I^131^ resistance	Modulate miR-9-5p/DPP4 axis	[244]
GAS5	Thyroid cancer	Promote proliferation and I^131^ resistance	Modulate miR-362-5p/SMG1 axis	[245]

## lncRNA biogenesis during pathological processes

### Transcriptional mechanisms

**Fig. 3:**
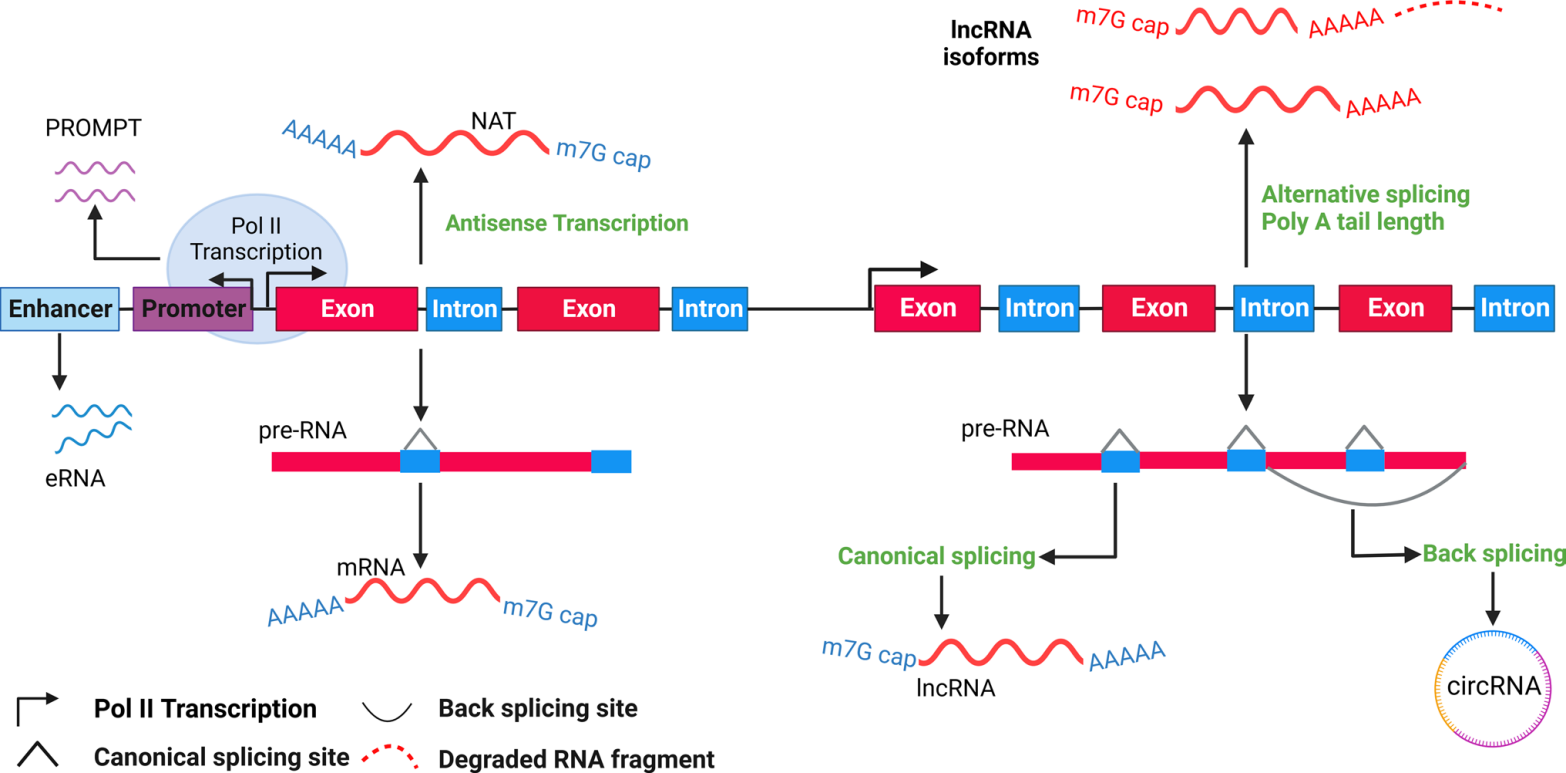
Processes governing the formation of lncRNAs and their positional categorizations based on genomic location concerning nearby protein-coding genes include bidirectional, intergenic, antisense, antisense intronic, sense intronic, enhancer, and sense-overlapping classifications

During pathological processes such as cancer, the landscape of lncRNAs undergoes significant changes, with specific classes of lncRNA emerging as key players in disease progression (Fig. 3). The abnormal regulation of transcription processes can lead to the production of lncRNAs closely associated with tumorigenesis and metastasis, known as oncogenic lncRNAs. These lncRNAs are instrumental in driving tumor growth and conferring resistance to therapeutic interventions [69–72]. This complex interplay between lncRNAs and the cellular machinery adds layers of regulation and functionality, underscoring the versatility of lncRNAs in gene expression and cellular dynamics. Some lncRNAs may not play direct functional roles but instead contribute to cellular processes as structural scaffolds or decoys. This lncRNA can influence gene expression indirectly by modulating the transcriptional landscape or affecting the stability of other RNA molecules.

### Posttranscriptional modifications

Furthermore, lncRNAs interact with various RNA modifications, notably N6-methyladenosine (m6A), which influences their stability, localization, and overall function within the cell. m6A modification, which occurs at specific genomic loci, plays a crucial role in the posttranscriptional regulation of lncRNAs, affecting their role in cancer and other diseases [73]. Recent studies have shed light on how the modulation of enzymes responsible for m6A methylation can significantly alter the biogenesis and functionality of lncRNAs, highlighting a subtle yet impactful mechanism of gene regulation with minimal effects on the coding transcriptome [74]. In some instances, lncRNAs are produced as a result of transcriptional noise, serving no clear benefit to the cell, yet their presence underscores the complexity of genomic transcription [33]. Despite the intricate nature of lncRNA biology and the challenges it presents, the critical role of lncRNAs in a wide array of diseases, particularly cancer, cannot be overstated. The involvement of lncRNAs in key cellular processes and disease mechanisms positions lncRNAs as promising targets for therapeutic intervention, offering new avenues for treatment strategies aimed at modulating their expression or function.

**Fig. 4:**
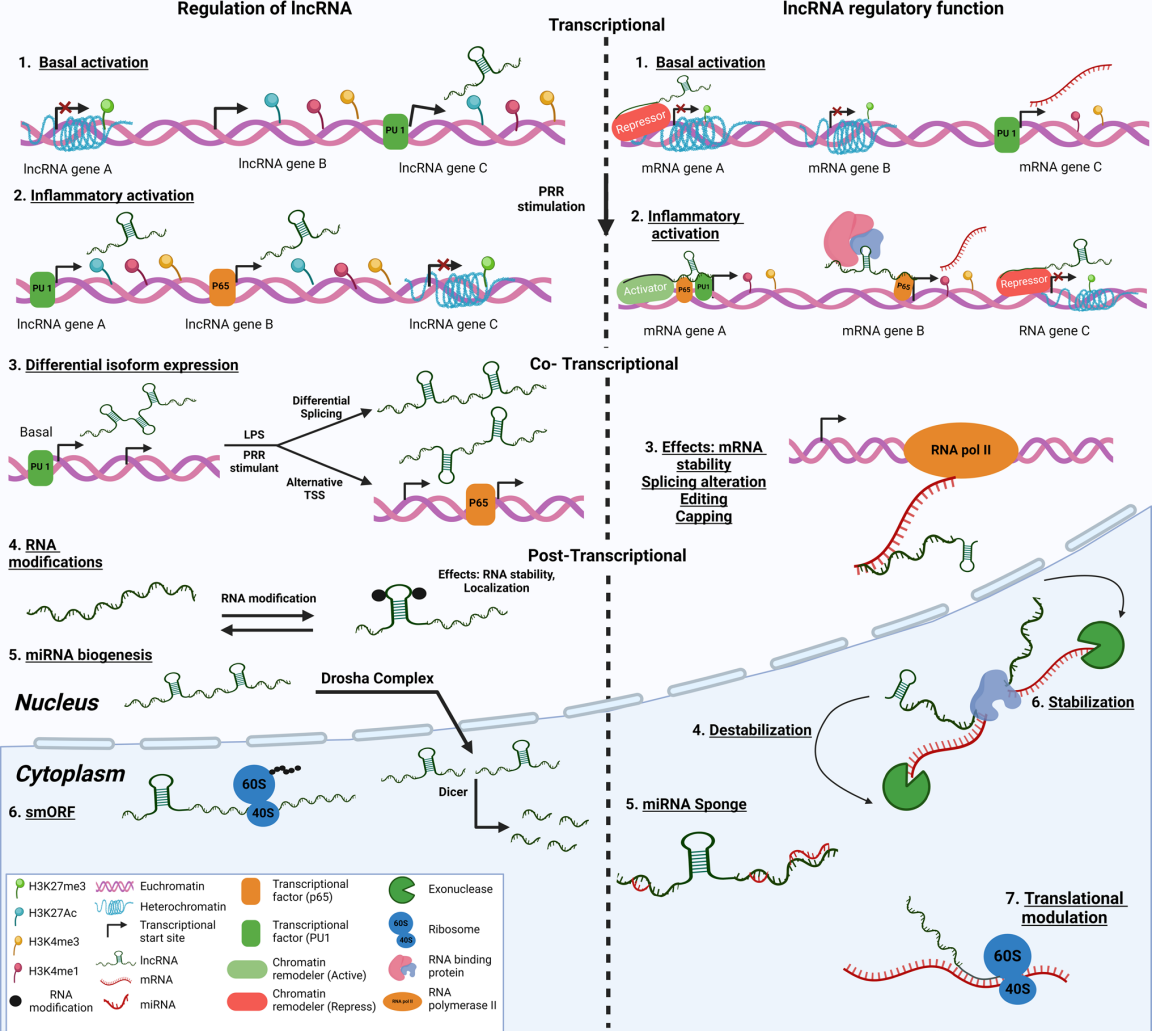
Regulation and classification of lncRNA. **A** The figure’s lower left section illustrates the transcriptional regulation mechanisms of lncRNA. It shows basal transcriptional activation ‘Part 1’, and its enhancement during inflammatory responses ‘Part 2’ triggered by the activation of pattern recognition receptors (PRR). It presents three lncRNA gene examples, labeled A, B, and C. ‘Part 3’ demonstrates the co-transcriptional regulation of lncRNA through differential isoform expression, which can involve alternative splicing or the employment of novel transcription start sites in response to inflammatory stimuli like lipopolysaccharides (LPS). The representation of post-transcriptional regulation of lncRNAs is categorized into three segments: 4, 5, and 6. Following transcription, lncRNAs undergo diverse processes. In ‘Part 4,’ RNA modifications are depicted, influencing the structural configuration of the lncRNA molecule. These modifications can be reversible, contingent upon the cellular inflammatory condition. ‘Part 5’ delineates the transformation of lncRNA into mature miRNA during miRNA biogenesis. ‘Part 6’ signifies the potential translation of lncRNAs containing small open reading frames (smORFs). **B** The lower right section of the figure illustrates the regulatory roles of lncRNAs within the nucleus and cytoplasm. It indicates that during transcription, both basal ‘Part 1’ and inflammatory ‘Part 2’ lncRNAs can either suppress (mRNA genes **A** and **C**) or enhance (mRNA genes **A** and **B**) gene expression

LncRNAs are categorized into five primary types based on their genomic location: antisense, bidirectional, intronic, enhancer-associated, and intergenic. Intergenic and enhancer-associated lncRNAs have distinct promoters and are independent of protein-coding genes. Conversely, bidirectional lncRNAs share a common promoter but are transcribed from the opposite strand of a protein-coding gene. Intronic lncRNAs originate within the introns of genes that encode proteins (Fig. 4).

### LncRNAs as regulators of transcription

By playing a crucial role in the extensive landscape of the human genome, lncRNAs function as transcriptional regulators, coordinating gene expression at the transcriptional level [75]. Their ability to modulate chromatin architecture and interact with transcription factors, DNA, and other molecular entities, such as RNA‒DNA hybrids (R-loops), positions them as key players in gene regulation [75–78]. This regulatory capacity is particularly evident in pathological states such as cancer, where aberrant lncRNA expression correlates with altered gene expression profiles, presenting new avenues for therapeutic intervention [79]. In the context of posttranscriptional regulation, lncRNAs act as miRNA sponges, influencing gene expression by sequestering miRNAs and preventing them from degrading target mRNAs. This mechanism is crucial in various cancers, underscoring the significance of lncRNA‒miRNA interactions in disease progression [80].

### LncRNAs as miRNA sponges

Recent studies have highlighted the crucial roles played by lncRNAs in cellular processes, with a particular emphasis on their role as miRNA sponges in cancer biology. The posttranscriptional modulation of gene activity by lncRNAs through miRNA sponging is a significant factor in various cancer types, underscoring the importance of the interplay between lncRNAs and miRNAs in tumorigenesis. Notably, the lncRNA UCA1, characterized by its structural stability attributed to specific genetic variations, has been linked to an elevated risk of endometriosis, a condition exhibiting notable parallels to cancer [81]. By functioning as a competing endogenous RNA (ceRNA), UCA1 hinders the activity of miRNAs, resulting in the dysregulation of downstream genes. Gene network analyses revealed alterations in UCA1 expression associated with pivotal pathways, such as fatty acid metabolism and mitochondrial beta-oxidation, in the context of endometriosis. In a parallel manner, LINC00662, which is upregulated in prostate cancer, serves as a miR-34a sponge, exerting influence on the progression of the disease [82, 83]. In hepatocellular carcinoma (HCC), ZFPM2-AS1 functions as a competing endogenous RNA (ceRNA), competitively binding with miR-139 and consequently influencing the expression of GDF10, a gene associated with the progression of HCC [84]. Research in breast cancer has focused on the involvement of miRNA sponges in the regulation of protein‒protein interactions (PPIs), emphasizing the essential role of interactions between lncRNAs and miRNAs in cancer [85].

### LncRNA as modulators of RNA stability

LncRNAs, including mRNAs and other noncoding RNAs, are instrumental in regulating the stability of RNA molecules. This regulatory function influences gene expression and impacts diverse cellular processes [86]. For instance, CiRS-7/CDR1as stabilizes its target mRNA through RNA duplex formation [87], and HNF1A-AS1 engages the ELAV1 protein to augment the stability and translation of particular mRNAs, such as CKAP5, involved in microtubule function and cancer cell cycle progression [88]. By forming an RNA‒protein complex with IGF2BP2, the lncRNA PCAT6 increases the stability of IGF1R mRNA, affecting IGF1R expression [89]. Additionally, the m6A modification of lncRNAs, as observed for HNF1A-AS1, alters their stability, revealing the nuanced regulation of gene expression via posttranscriptional modifications [88].

## LncRNA and RBP interactions

In the regulatory landscape, lncRNAs serve as crucial partners for RNA binding proteins (RBPs), guiding their localization and function within cells [90]. This interaction allows lncRNAs to influence cellular processes significantly, from modulating the nuclear translocation of proteins to acting as scaffolds for complex molecular structures [91, 92]. Notably, HuR is upregulated in cancer and plays a pivotal role in disease progression by post-transcriptionally regulating cancer-related mRNAs [93, 94]. It enhances the stability of NEAT1 in ovarian cancer, influencing crucial cancer pathways as a ceRNA [95], and stabilizes lncRNA-HGBC in gallbladder carcinoma to promote cell proliferation and invasion [96]. Conversely, HuR can degrade lncRNAs such as p53-regulated lncRNA-p21, demonstrating its context-dependent dual role in RNA stability [97]. Similarly, serine/arginine-rich splicing Factor 1 (SRSF1) is implicated in glioma, where its knockdown alters mRNA and lncRNA expression, notably downregulating NEAT1, confirming its role in maintaining NEAT1 levels [98]. This finding indicates the nuanced roles of RBPs such as SRSF1 and HuR in modulating lncRNA stability, which impacts cancer progression [95, 98]. The AUF1 complex, which arises from alternative splicing of the HNRNPD gene, also plays a significant role in posttranscriptional lncRNA regulation [99]. It competes with HuR to bind U-rich sequences of AREs, influencing the decay or stabilization of lncRNAs such as NEAT1, and the effects of MALAT1 AUF1 vary, destabilizing NEAT1, which disperses nuclear paraspeckles and affects mRNA export; however, it does not impact MALAT1 stability, underscoring the complexity of its lncRNA interactions [100]. PABPN1, another nuclear RBP, binds the 3ʹ poly(A) tail of RNA, influencing the stability of lncRNAs such as NEAT1 and TUG1. The depletion of PABPN1 in HeLa cells impacts specific polyadenylated lncRNAs, indicating its role in sophisticated regulatory networks involving the nuclear exosome [101, 102]. IGF2BP1, which is frequently reactivated in cancers such as HCC, interacts with the lncRNA HULC to uniquely promote its degradation, a departure from its usual stabilizing role with mRNAs. This interaction involving the CCR4-NOT complex highlights the impact of structural motifs and protein partners on RBP–lncRNA interactions [103]. Tristetraprolin (TTP), unlike HuR and AUF1, generally acts as a tumor suppressor by destabilizing oncogenic RNAs such as HOTAIR. Its downregulation in cancer cells leads to reduced RNA degradation, underscoring the importance of RBPs such as TTP in cancer progression and their potential as therapeutic targets [104, 105]. The binding of lncRNAs to RBPs can alter the function of these proteins, influencing mRNA stability and translation, RNA splicing, and other critical regulatory processes. In cancer, these interactions often lead to changes in cell proliferation, apoptosis, and metastasis, making them potential targets for therapeutic intervention.

## LncRNAs as translated peptides

Challenging the traditional view of lncRNAs as merely noncoding RNAs, recent studies have revealed their potential to engage with ribosomes and translate into functional peptides [106, 107]. This novel understanding, supported by comprehensive studies such as those by L. Minati et al. and ribosome profiling efforts by Bernardo Bonilauri, opens up exciting possibilities for lncRNA functionality beyond mere transcriptional regulation. The identification of lncRNAs with small open reading frames (smORFs) suggests a hidden layer of proteomic complexity and functional diversity within the realm of noncoding RNAs [108, 109]. Among these, several lncRNAs were found to contain small open reading frames (smORFs) that potentially encode functional microproteins. In colorectal cancer research, the lncRNA EVADR, influenced by *Fusobacterium nucleatum* infection, has been shown to play a role in cancer metastasis by acting as a scaffold for YBX1 and promoting EMT-related translation [110]. The development of tools such as LncDC by Minghua Li and Chun Liang further enhanced our ability to uncover lncRNAs with translational capabilities, suggesting that a subset of these molecules may indeed encode functional peptides, challenging their conventional classification and highlighting their potential in novel therapeutic strategies [107, 111, 112].

## Challenges and approaches in identifying and characterizing lncRNAs

The integration of lncRNAs in clinical practice for cancer treatment necessitates accurate RNA analysis to identify and quantify novel RNA species. The unique sequences of lncRNAs and their potential overlap with other RNA types present significant challenges. For example, the HOTTIP lncRNA has been identified as a key regulator in gastrointestinal cancers, presenting new opportunities for diagnosis and treatment. Furthermore, research into plant stress responses and colorectal cancer has underscored the importance of lncRNAs in elucidating disease mechanisms and their utility as biomarkers in exosomes for early detection and intervention [113, 114].

### Techniques for identifying lncRNAs

The landscape of transcriptomics has been transformed by RNA sequencing (RNA-seq) and other high-throughput sequencing technologies. These advancements allow for comprehensive annotation and quantification of a diverse range of RNAs, encompassing both coding and noncoding transcripts [115]. The selective extraction of polyadenylated RNA, excluding ribosomal RNA (rRNA), has been instrumental in isolating lncRNAs [116]. Continued progress, including the creation of libraries depleted of rRNA and the implementation of random priming during cDNA synthesis, has enhanced the precision of characterizing lncRNAs [117]. Traditional methods, including RT‒qPCR and Northern blotting, are fundamental for the validation and quantification of specific lncRNAs [118]. Given the vast diversity of lncRNAs, achieving specificity in their study is crucial. Techniques employing RNAe H to target RNA‒DNA hybrids have been developed to identify particular lncRNAs within complex RNA landscapes [119]. Microarray analysis, using probes designed for specific lncRNA sequences, is an initial screening tool, although its results require rigorous validation due to potential inconsistencies [120]. The visualization and quantification of lncRNAs within cells are crucial for comprehending their biological functions. Techniques such as RNA fluorescence in situ hybridization (RNA-FISH) are employed to evaluate the spatial distribution of lncRNAs, revealing their interactions with proteins and miRNAs. This approach offers valuable insights into the functional mechanisms of lncRNAs [121]. Additionally, RNA-FISH has played a crucial role in revealing the interactions between lncRNAs and other cellular components, including proteins and miRNAs [122]. Recent progress has resulted in the creation of assays characterized by improved sensitivity and specificity. Notably, reverse transcription-droplet digital polymerase chain reaction (RT-ddPCR) is a robust technique for accurately quantifying even low-abundance lncRNAs. Additionally, the rolling circle amplification (RCA) technique represents another innovative method that facilitates the amplification of specific lncRNA sequences to achieve clear and precise detection [123].

### Approaches for the functional assessment of lncRNAs

Beyond detection, understanding the function of lncRNAs requires a comprehensive approach. lncRNAs interact with a broad spectrum of molecular entities, including DNA, RNA, microRNA (miRNA), and proteins, thereby influencing gene expression and cellular signaling pathways [124]. For instance, the lncRNA GAS5 impacts hepatic lipid metabolism, with its knockdown resulting in a reduction in lipid accumulation, suggesting its potential as a target for treating conditions such as NAFLD [125]. Similarly, the lncRNA MG828507, located upstream of the FLT1 gene, has been associated with preeclampsia, highlighting its significance in disease pathogenesis [126]. Computational tools have become invaluable in elucidating lncRNA functions, especially in mapping lncRNA interactions with proteins and RNA. These tools utilize a range of models, from ensemble-based and machine-learning approaches to molecular docking and network analyses, facilitating a deeper understanding of lncRNA roles [127]. Cutting-edge sequencing technologies, such as Oxford Nanopore, provide thorough insights into the sequences of lncRNAs, while NanoString platforms allow precise detection and quantification without the necessity for amplification or reverse transcription [128, 129].

## LncRNA as potential biomarkers

**Fig. 5:**
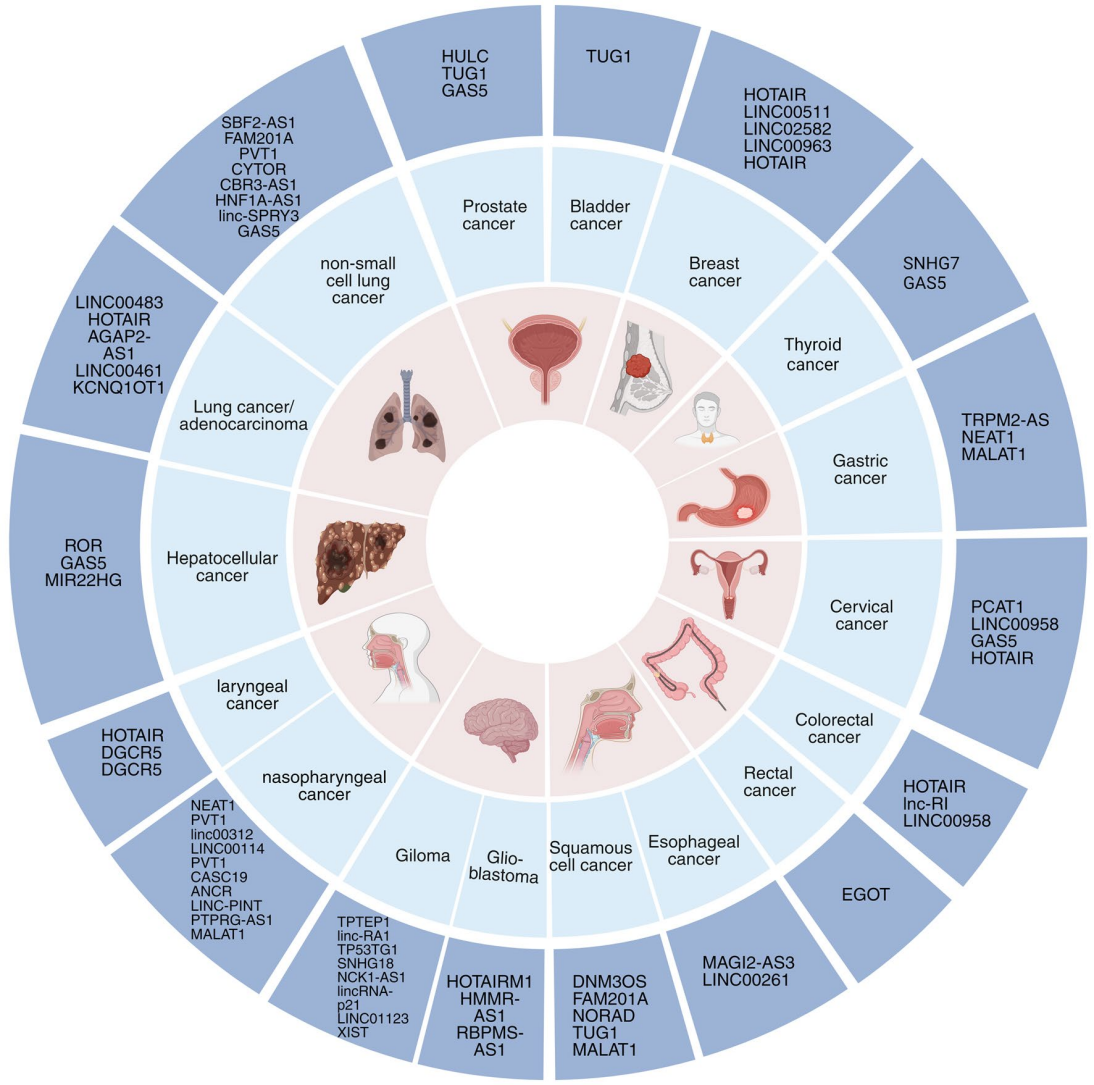
LncRNA as biomarkers and therapeutic agents in human oncology. A contemporary overview of lncRNA with potential as clinical biomarkers (outermost layer) and/or as targets for therapy (represented by syringe symbols directed at the diagram), linked to various cancer forms

The roles of lncRNAs in numerous physiological processes, such as cell differentiation and proliferation, are gaining increasing recognition. This finding positions them as significant contributors to the development and progression of cancer [130]. Their association with specific clinical features, such as tumor grade, size, metastasis stage, and overall aggressiveness, underscores their potential as biomarkers for cancer [131]. The distinct expression profiles, stability, specificity, and distribution of lncRNAs enable their identification and measurement in bodily fluids through liquid biopsy. This method provides a noninvasive approach for early cancer detection, diagnosis, prognosis, and monitoring of therapeutic responses (Fig. 5). Ongoing research has revealed the potential of lncRNAs as liquid-based diagnostic biomarkers, particularly for head and neck cancer (HNC) and other malignancies [132]. Cutting-edge sequencing technologies, such as Oxford Nanopore, offer an in-depth understanding of lncRNA sequences. Moreover, NanoString platforms enable the accurate detection and quantification of these sequences without the necessity for amplification or reverse transcription [133].

Certain lncRNA have shown significant prognostic value across various cancers. For instance, in gastric cancer, early detection linked to specific lncRNA profiles correlates with improved 5-year survival rates [134]. In colorectal cancer (CRC), a 3-lncRNA signature has been identified as a potential prognostic marker, emphasizing the role of lncRNAs in predicting disease progression and outcomes. Research at the Oncology Institute of Porto into lncRNA/miRNA ceRNA networks highlights their importance in cancer prognosis [135, 136]. Pancancer analyses have revealed the prognostic significance of lncRNAs, positioning them as crucial elements in cancer research and potential therapeutic targets [137]. In breast cancer, the analysis of lncRNA-TF-associated ceRNA networks has led to the identification of novel prognostic biomarkers, illustrating the intricate interplay between lncRNAs and other cellular components in cancer progression [138].

## lncRNA as novel cancer biomarkers

### Biomarkers for diagnosis

LncRNA has emerged as a significant biomarker in cancer, offering insights into diagnosis, prognosis, and the prediction of therapeutic responses. The increasing mortality rate from cancer has intensified the search for reliable diagnostic tools, with circulating lncRNAs showing considerable promise. These molecules are being investigated for their potential to detect various cancers early, including hepatocellular carcinoma, colorectal cancer, gastric cancer, renal cell carcinoma, and prostate cancer, through liquid biopsies [132, 139–142]. Prostate cancer antigen 3 (PCA3) has become an instrumental biomarker for prostate cancer, notably enhancing early detection and screening accuracy beyond traditional prostate-specific antigen (PSA) testing [143]. The higher specificity of PCA3 helps mitigate the frequency of unnecessary biopsies, presenting a significant advantage over PSA tests, whose levels may vary with prostate size. [144, 145]. Notably, PCA3 is the first long noncoding RNA biomarker to gain FDA approval for cancer screening, establishing a groundbreaking precedent in cancer diagnostics [144, 146]. In practice, PCA3 is quantified from urine samples obtained post-digital rectal exam, offering a noninvasive assessment tool for men with elevated PSA levels [145]. The integration of PCA3 into existing diagnostic frameworks is continually advancing, with research efforts focused on improving diagnostic accuracy by combining it with other markers and imaging techniques. Additionally, its potential in personalized medicine is being explored to tailor treatment strategies based on individual biomarker profiles.

### Biomarkers for prognosis

Specifically, the lncRNA HOTAIR has been identified as a crucial marker in glioma, where its elevated expression correlates with higher disease grades and poorer patient outcomes, highlighting its role in both diagnosis and prognosis [147]. In addition to their diagnostic utility, lncRNAs serve as valuable prognostic indicators, with HOTAIR standing out for its independent prognostic value in glioma. This finding not only suggests the potential for targeted therapeutic strategies but also underscores the broader applicability of lncRNAs in predicting disease progression. Furthermore, a deep learning study demonstrated the effectiveness of specific lncRNAs in predicting significant disease associations, reinforcing their role in prognosis [147, 148].

## LncRNAs as novel therapeutic targets

### Inhibiting oncogenic lncRNAs

The modulation of lncRNA expression through RNA interference (RNAi) techniques, such as the use of small interfering RNA (siRNA) or short hairpin RNA (shRNA), offers a targeted approach to silence oncogenic lncRNAs in vivo. For instance, targeting HOTAIR in breast cancer and MALAT1 across various cancers has demonstrated potential in reducing tumor growth and improving prognosis [149, 150]. Antisense oligonucleotides (AONs) further enable the selective inhibition of lncRNAs such as GAS5, showing efficacy in tumor reduction [151].

### Enhancing tumor-suppressive lncRNAs

Conversely, strategies to upregulate lncRNAs, particularly those that act as miRNA sponges or competing endogenous RNAs (ceRNAs), have been explored to counteract oncogenic miRNAs. Techniques employing lentivirus or adeno-associated virus vectors and nanoparticle encapsulation have facilitated the overexpression of lncRNAs such as GAS5 and UCA1, sensitizing cancer cells to radiation or chemotherapy and offering new avenues for overcoming treatment resistance [151–153].

### Combining RNA therapy with conventional therapies

Advances in delivery methods, including the use of nanoparticles such as gold nanoparticles (AuNPs), have enhanced the efficiency of RNAi and AONs in targeting lncRNAs such as NEAT1 and XIST, contributing to tumor growth inhibition and increased survival in preclinical models [154, 155]. Additionally, drug inhibitors targeting lncRNAs such as ANRIL have shown promise in diminishing tumorigenicity, highlighting the therapeutic potential of modulating lncRNA activity [156]. In gastric cancer, the lncRNA CRNDE has been recognized as a crucial modulator of autophagy-related chemoresistance. Elevated CRNDE levels sensitize gastric cancer cells to chemotherapy by suppressing autophagy. Targeting the E2F6-CRNDE axis has emerged as a potential therapeutic strategy against chemoresistance in gastric cancer [157].

## Overcoming treatment resistance and sensitivity through lncRNA modulation

The expression of lncRNAs is a key factor in cancer treatment resistance. LncRNAs such as H19 and UCA1 have been implicated in chemoresistance mechanisms in ovarian and gastric cancers, respectively, through their interaction with miRNAs and signaling pathways [158, 159]. Targeting these lncRNAs offers a strategy to enhance the efficacy of conventional therapies, such as cisplatin and tamoxifen, and improve patient outcomes. Moreover, the role of lncRNAs in promoting drug resistance through exosomal transfer, as observed with TP73-AS1 in glioblastoma and its contribution to temozolomide resistance, emphasizes the complexity of cancer biology and the potential of lncRNAs as therapeutic targets [160–162]. In cancer therapy, lncRNAs are pivotal for understanding treatment resistance. The expression profiles of these genes can change in response to chemotherapy, radiotherapy, and immunotherapy, affecting treatment outcomes [163]. For example, the lncRNA SNHG6, which acts as a miR-101 sponge, promotes epithelial–mesenchymal transition (EMT), a process linked to metastasis and resistance, particularly by influencing the responsiveness of breast cancer to tamoxifen [164]. Similarly, in colorectal cancer, UCA1 has been shown to confer resistance to cetuximab by modulating the miR-495 and HGF/c-MET signaling pathways, indicating its role in therapeutic resistance [165]. Prostate cancer cells resistant to androgen receptor inhibitors such as enzalutamide exhibit altered lncRNA expression patterns, suggesting a mechanism for the development of resistance [166]. In chemotherapy-sensitive lung cancer tissues, the lncRNA MEG3 is expressed at lower levels, and its overexpression reduces autophagy levels, thereby enhancing the effectiveness of vincristine in lung cancer chemotherapy [167]. Reducing the expression of the lncRNA HOXD-AS1 in glioma cells decreased their proliferation, migration, and invasion while increasing their sensitivity to cisplatin (DDP). This effect is mediated through the sequestration of miR-204, suggesting that HOXD-AS1 could serve as a viable therapeutic target for glioma treatment [168].

## Conclusion and future prospects for lncRNA research

Advances in sequencing technologies have significantly refined our understanding of lncRNAs, increasingly distinguishing functional molecules from mere transcriptional noise. This progress, however, underscores the need for rigorous validation to confirm the biological roles of lncRNAs, with current guidelines aiming to streamline these efforts. Emerging at the forefront of cancer research, lncRNAs offer promising avenues for diagnostic and therapeutic innovations due to their involvement in key physiological processes. However, their full impact on cancer progression and utility in clinical settings warrants careful scrutiny, especially considering the potential unintended effects in healthy tissues and the complexity of their molecular pathways. The evolution of lncRNA research necessitates rigorous biological validation using patient samples alongside comprehensive functional studies to harness their potential effectively. Enhancing the sensitivity, specificity, and accuracy of lncRNA biomarkers by employing advanced molecular tools such as RNA-seq, RNA-FISH, ic-SHAPE, and quantitative real-time PCR is crucial. These techniques are essential for accurately measuring lncRNA levels in biological samples, providing insights into their roles in health and disease. Moreover, the safety, toxicity, and side effects associated with lncRNA-targeted therapies require thorough evaluation. Innovations in delivery methods, such as nanoparticle-based systems, and the integration of RNA therapy with other treatments are critical to advancing therapeutic efficacy and improving patient outcomes. Additionally, employing computational methods to understand how lncRNAs predict disease associations is pivotal, utilizing machine learning and other data-driven approaches to model lncRNA interactions and their functional impacts. Although lncRNAs hold significant promise for revolutionizing cancer diagnosis and therapy, realizing this potential involves overcoming numerous challenges. By focusing on these strategic areas, we aim to inspire ongoing research and innovation within the field, advancing toward effective clinical applications of lncRNAs.

## Data Availability

Not applicable.
